# Adolescents’ first tobacco products: Associations with current multiple tobacco product use

**DOI:** 10.1371/journal.pone.0217244

**Published:** 2019-05-23

**Authors:** Sarah D. Kowitt, Adam O. Goldstein, Erin L. Sutfin, Amira Osman, Clare Meernik, Courtney Heck, Leah M. Ranney

**Affiliations:** 1 Department of Family Medicine, School of Medicine, University of North Carolina at Chapel Hill, Chapel Hill, NC, United States of America; 2 Lineberger Comprehensive Cancer Center, Chapel Hill, NC, United States of America; 3 Department of Social Sciences and Health Policy, Wake Forest School of Medicine, Winston-Salem, NC, United States of America; 4 School of Nursing, Zefat Academic College, Zefat, Israel; 5 Department of Epidemiology, Gillings School of Global Public Health, University of North Carolina at Chapel Hill, Chapel Hill, NC, United States of America; 6 North Carolina Department of Health and Human Services, Tobacco Prevention and Control Branch, Raleigh, NC, United States of America; Medical University of South Carolina, UNITED STATES

## Abstract

Understanding which tobacco products adolescents use first can lead to insights for tobacco prevention interventions and policies. We used cross-sectional data from high school students who reported ever using a tobacco product from the 2017 North Carolina Youth Tobacco Survey (n = 1,053). In multivariable regressions, we examined how demographic and psychosocial factors were associated with adolescents’ first product tried and how first product tried was associated with current tobacco use (i.e., no use, use of a single product, use of multiple products) and frequency of tobacco use. Cigarettes (34.8%) and e-cigarettes (33.7%) were the most frequently reported first products tried, followed by cigars (15.6%), smokeless tobacco (10.7%), waterpipe (4.0%), and other tobacco products (i.e., pipe tobacco or some other tobacco product) (1.2%). Demographic differences in adolescents’ first product tried existed, with Black adolescents having higher odds of initiating tobacco use via cigars (aOR: 6.17, 95% CI: 3.75, 10.14). Adolescents who initiated tobacco use via cigars (aOR: 2.33, 95% CI: 1.31, 4.13) or smokeless tobacco (aOR: 2.45, 95% CI: 1.18, 5.04) had higher odds of being a multiple current tobacco product user, whereas adolescents who initiated tobacco use via e-cigarettes (aOR: 0.57, 95% CI: 0.34, 0.93) had lower odds of being a multiple current tobacco product user. Additionally, adolescents who initiated tobacco use via smokeless tobacco had higher odds of currently using at least one tobacco product frequently (aOR: 1.90, 95% CI: 1.04, 3.48), while adolescents who initiated tobacco use via e-cigarettes had lower odds of currently using at least one tobacco product frequently (aOR: 0.40, 95% CI: 0.23, 0.70). These findings suggest that most adolescents reported initiating tobacco use via cigarettes or e-cigarettes and that trying certain products first (e.g., cigars, smokeless tobacco) was associated with higher odds of multiple current tobacco product use.

## Introduction

Over the last decade, the tobacco product landscape has changed markedly. Given these changes, data on how adolescents initiate tobacco use (i.e., which products they try first and demographic factors associated with first products tried) is needed. These data are important for several reasons. First, all tobacco products contain nicotine, which is highly addictive. Nicotine causes harm to adolescents’ developing brains and can lead to future tobacco use [1]. To prevent adolescents’ exposure to nicotine and tobacco products, we need to know how adolescents are initiating tobacco use (i.e., which products they are trying first). Second, in order to effectively prevent tobacco product use, we need to know how demographic factors are associated with which products adolescents try first. For instance, if Black adolescents are typically only trying cigars first, then interventions to prevent cigar use can be tailored to Black adolescents. Finally, if data indicate that trying certain products first (e.g., cigarettes) is associated with adolescents currently using multiple tobacco products, then tobacco prevention interventions and policies can be targeted toward those products.

While historically tobacco use has begun with cigarettes [2–4], recent data suggest that the landscape for tobacco product initiation may be changing. One paper, using national data from 2014–2015, found that 18.6% of adolescents reported ever trying a tobacco product, and while cigarettes were the most frequently reported first product tried, sizeable proportions of adolescents reported trying e-cigarettes or smokeless tobacco (SLT) first [5]. This study also found demographic differences in first product tried. Specifically, females (relative to males) and Hispanic adolescents (relative to non-Hispanic adolescents) were more likely to initiate tobacco use via cigarettes; White adolescents (relative to non-White adolescents) were more likely to initiate tobacco use via SLT; and adolescents of higher socioeconomic status (SES) were more likely to initiate tobacco use via waterpipe (relative to those of lower SES) [5]. Three other studies have examined adolescents’ first products tried [6–8] but only examined certain tobacco products (i.e., cigars and cigarettes with data from 2003–2004 [7]); only examined first product tried in a limited sample (i.e., adolescent lifetime e-cigarette users in Connecticut [6]); or examined first product tried in a sample of both adolescents and young adults, but did not differentiate results by age group [8].

Further, it is important to examine whether trying certain products first is associated with higher risk of multiple tobacco product use (i.e., concurrent use of two or more tobacco products) and higher risk of using tobacco products frequently. Over two million adolescents, almost half of adolescent tobacco users, are multiple tobacco product users [9]. Multiple tobacco product use is concerning because it is associated with increased symptoms of nicotine dependence [10], elevated risk of health concerns (e.g., myocardial infarction) [11], and decreased likelihood of successful quitting [12, 13]. While certain demographic and psychosocial factors are associated with increased risk for multiple tobacco product use (e.g., being male, having a higher propensity for sensation-seeking, having tried tobacco at a younger age) [8], little research has examined if *trying* certain tobacco products first is associated with multiple tobacco product use. If research shows that initiating tobacco product use via certain products is associated with greater risk of multiple tobacco product use, then interventions can be targeted toward those specific products. Although some research has shown that initiating e-cigarette use is associated with future tobacco use [14–18], research has not differentiated whether trying certain tobacco products first (relative to other tobacco products) is associated with current multiple tobacco use. To our knowledge, no studies have examined how first product tried is associated with past 30 day (current) multiple tobacco product use or with frequency of tobacco product use in the past 30 days.

Our study had three aims: 1) examine how demographic and psychosocial factors are associated with which tobacco products adolescents try first; 2) examine how first product tried is associated with current tobacco product use (e.g., no use, single tobacco product use, multiple tobacco product use); and 3) examine how first product tried is associated with frequency of tobacco product use (e.g., frequent or non-frequent tobacco product use). We hypothesized, given recent data in rising prevalence of e-cigarettes [19], that many adolescents would report trying e-cigarettes first. Consistent with previous research [5], we also hypothesized that demographic and psychosocial factors would be associated with which products adolescents try first. Finally, given a lack of previous research, we had no a priori hypotheses on whether or not trying certain products first would be associated with current tobacco product use or frequency of tobacco product use.

## Methods

### Participants and procedures

We used data from the 2017 North Carolina Youth Tobacco Survey (NCYTS). The NCYTS is a public and charter school-based survey of students in grades 6–12 and designed to provide data on tobacco use and progress toward overall goals among youth. A multi-stage cluster sampling design in three distinct regions of the state was used. School districts were first selected within three geographic regions of the state; a school’s probability for selection was proportional to its enrollment size for the survey year. Classes were then randomly selected within each school. Participation was voluntary and anonymous. Passive consent forms were utilized, unless an active consent form was required according to a specific school district policy. We focused our analyses on high school students given low rates of tobacco use among middle school students. The overall response rate was 64.5% for high school students (75.2% school response rate; 85.8% student response rate). With regard to ethical approval, our study used secondary, de-identified data and did not constitute human subjects research as defined under federal regulations 45 CFR 46.102 (d or f) and 21 CFR 56.102(c)(e)(l); hence, this study did not require Institutional Review Board approval.

### Measures

All measures described below can be seen in the 2017 NCYTS questionnaire, which is published online and accessible using this website: https://www.tobaccopreventionandcontrol.ncdhhs.gov/data/yts/docs/2017-NC-YTS-Questionnaire-FINAL.pdf.

#### First tobacco product tried

Our main outcome of interest was first tobacco product tried, which the survey assessed by asking participants, “Which of the following tobacco products did you try first (choose only one answer).” Responses included: 1) cigarettes, 2) cigars (including little cigars and cigarillos), 3) tobacco in a hookah or a waterpipe, 4) pipe tobacco, 5) electronic cigarettes, 6) chewing tobacco, snuff, or dip, 7) snus, and 8) dissolvable tobacco, 9) some other tobacco product, 10) not sure about the product I tried first, and 11) I have never tried any tobacco products. We removed participants who responded that they were not sure about the product that they tried first or reported never trying any tobacco products.

We collapsed responses of chewing tobacco, snuff, dip, snus, and dissolvable tobacco into one category and labeled it SLT. Due to small sample sizes, we also collapsed responses of pipe tobacco and “some other tobacco product” into one category and labeled it “other tobacco products” (OTPs).

#### Current multiple tobacco product use

Our second outcome of interest was current multiple tobacco product use. The survey assessed past 30 days use of nine tobacco products, including those mentioned above and bidis. Adolescents were then classified as 1) non-current tobacco product users if they did not use any tobacco product within the past 30 days, 2) single current tobacco product users if they used only one tobacco product within the past 30 days, and 3) multiple current tobacco product users if they indicated using two or more tobacco products within the past 30 days.

#### Frequency of tobacco product use

Our third outcome of interest was frequency of tobacco product use among current users. The survey assessed frequency of tobacco use of five different tobacco products: cigarettes, cigars, e-cigarettes, SLT, and waterpipe. The question stem was “During the past 30 days, on how many days did you smoke…? Responses included “0 days,” “1 or 2 days,” “3 to 5 days,” “6 to 9 days,” “10–19 days,” “20 to 29 days,” and “all 30 days.” In line with previous research [20], if participants chose “20 to 29 days” or “all 30 days,” they were coded as frequent users. Otherwise, participants were coded as non-frequent users if they chose any other response option.

#### Psychosocial variables

Psychosocial variables included: exposure to tobacco advertising via the Internet and retail locations, perceived risks of tobacco use, exposure to secondhand smoke, and living with tobacco product users. The survey assessed exposure to tobacco advertising via the Internet by asking participants, “When you are using the Internet, how often do you see ads for tobacco products, including electronic cigarettes?” In line with previous research [21], we dichotomized exposure as “yes” for responses “always,” “most of the time,” and “sometimes” and “no” for “I do not use the Internet,” “never,” and “rarely.” The survey assessed exposure to tobacco advertising via retail locations by asking participants, “When you go to a convenience store, supermarket, or gas station, how often do you see ads for tobacco products, including electronic cigarettes?” We dichotomized exposure to tobacco advertising via retail locations in the same way as we dichotomized exposure to tobacco advertising via the Internet.

The survey assessed perceived risks of tobacco use by asking participants two questions. First, participants were asked, “How strongly do you agree with the statement ‘All tobacco products are dangerous’?” In line with previous research [21], we dichotomized perceived risk as “yes” for the response “strongly agree” and “no” for the responses “agree,” “disagree,” and “strongly disagree.” Second, participants were asked, “Do you think that breathing smoke from other people’s cigarettes or other tobacco products is …” We dichotomized perceived risk as “yes” for the response “very harmful to one’s health” and “no” for the responses “somewhat harmful to one’s health,” “not very harmful to one’s health,” and “not harmful at all to one’s health.”

The survey assessed secondhand smoke exposure by asking participants, “During the past 7 days, on how many days did you breathe the smoke from someone who was smoking tobacco products in an indoor or outdoor public place? Examples of indoor public places are school buildings, stores, restaurants, and sports arenas. Examples of outdoor public places are school grounds, parking lots, stadiums and parks.” We dichotomized exposure as “yes” for the responses “1 day,” “2 days,” “3 days,” “4 days,” “5 days,” or “6 days” and “no for the response “0 days.”

The survey assessed living with a tobacco user by asking participants, “Does anyone who live with you now…” Response options included, “smoke cigarettes,” “smoke cigars, cigarillos, or little cigars,” “use chewing tobacco, snuff, or dip,” “use e-cigarettes,” “smoke tobacco in a hookah or waterpipe,” “smoke pipes filled with tobacco (not waterpipe),” “use snus,” “use dissolvable tobacco products,” “smoke bidis,” and “no one who lives with me now uses any form of tobacco.” We dichotomized living with a tobacco user as “yes” if participants chose any of the 9 listed tobacco products and “no” if participants chose “no one who lives with me now uses any form of tobacco.”

#### Demographics

Demographic variables included sex (female or male), grade (9^th^, 10^th^, 11^th^, or 12^th^), race/ethnicity (non-Hispanic White, non-Hispanic Black, Hispanic, or non-Hispanic other race), and adolescent socioeconomic status (SES). We measured adolescent SES by asking, “Do you get free or reduced-price lunch at school?” (yes or no) [22].

### Study analytic sample

Since we were interested in what factors were associated with adolescents’ first product tried, we restricted analyses to adolescents who had ever tried one of the nine listed tobacco products (“ever tobacco product users”). Of the 3,133 high school students in the 2017 NCYTS, we dropped participants who had never tried a tobacco product (n = 1,528), did not report their first product tried (n = 422), or who had missing data on any of the other study variables (n = 163), resulting in a final analytic sample of 1,020 participants (see S1 Table for an attrition analysis).

### Data analysis

To answer our first research question, we examined demographic and psychosocial correlates of first product tried using bivariate logistic regressions. Given the number of demographic and psychosocial correlates we were examining, we only entered correlates that were statistically significant (p <0.10) into a multivariable logistic regression model. For each tobacco product, adjusted odds ratios (aORs) and 95% confidence intervals (CIs) for using that product first versus any of the other tobacco products are presented.

To answer our second research question (whether first product tried is associated with current tobacco use), we examined which products adolescents were currently using by calculating weighted percentages of current tobacco product use stratified by first product tried. We then used a multivariable, multinomial logistic regression model and controlled for demographic correlates. Although our outcome, current tobacco use, was ordinal in nature, we specified a multinomial model with non-current tobacco users as the reference category because 1) the proportional odds assumption failed, which indicates that the effects of the covariates across the current tobacco use categories are not the same [23] and 2) it was of interest to compare multiple current tobacco product users to non-current tobacco product users and single current tobacco product users to non-current tobacco product users which has been done in previous studies [24]. Finally,

To answer our third research question (whether first product tried is associated with frequency of tobacco use among current users of any tobacco product), we used bivariate logistic regressions and a multivariable logistic regression model that controlled for demographic correlates. The outcome was whether participants frequently used any tobacco product vs. whether participants did not frequently use any tobacco products. All analyses used SAS version 9.4 survey procedures (SAS Inc., Cary, NC, USA). We set critical α = .05 (except as noted above) and used 2-tailed statistical tests.

## Results

### Participant characteristics

Among the 1,020 participants who reported ever tobacco product use, the majority identified as non-Hispanic White (64.0%) and almost half reported receiving free or reduced-price lunch (45.9%) (see Table 1). In this sample of ever tobacco product users, almost two-thirds (64.6%) reported they currently used a tobacco product, with 34.1% classified as single tobacco products users and 30.5% classified as multiple tobacco product users. Among current users of any tobacco product, 26.9% reported frequently using at least one tobacco product, with the remainder reporting non-frequent use (73.1%).

**Table 1:** Characteristics of ever tobacco product users, n = 1020, North Carolina Youth Tobacco Survey, 2017.

Characteristic	Total n (%)
Sex	
Female	492 (49.2)
Male	528 (50.8)
Grade	
9^th^	253 (20.4)
10^th^	261 (25.9)
11^th^	211 (26.2)
12^th^	295 (27.4)
Race	
Non-Hispanic White	612 (64.0)
Non-Hispanic Black	190 (17.9)
Hispanic	163 (11.0)
Non-Hispanic other race	55 (7.1)
Free or reduced-price lunch	
Yes	496 (45.9)
No	524 (54.1)
Exposure to tobacco advertising via the Internet	
No	514 (50.8)
Yes	506 (49.2)
Exposure to tobacco advertising via retail locations	
No	177 (15.9)
Yes	843 (84.1)
Perceived risk: Agreed that “all tobacco products are dangerous”	
No	664 (65.3)
Yes	356 (34.7)
Perceived risk: Agreed that “breathing smoke from other people’s cigarettes or other tobacco products is harmful”	
No	541 (57.2)
Yes	479 (42.8)
Exposed to secondhand smoke exposure	
No	525 (50.7)
Yes	495 (49.3)
Living with a tobacco product user	
No	422 (39.8)
Yes	598 (60.2)
Current multiple tobacco product use*	
No current use	380 (35.4)
Current single product use	312 (34.1)
Current multiple product use	328 (30.5)
Frequency of any tobacco product use among current users of any tobacco product	
Non-frequent	450 (73.1)
Frequent	187 (26.9)

* When collapsed, 64.6% of the sample was classified as a current tobacco product user.

**Fig 1:**
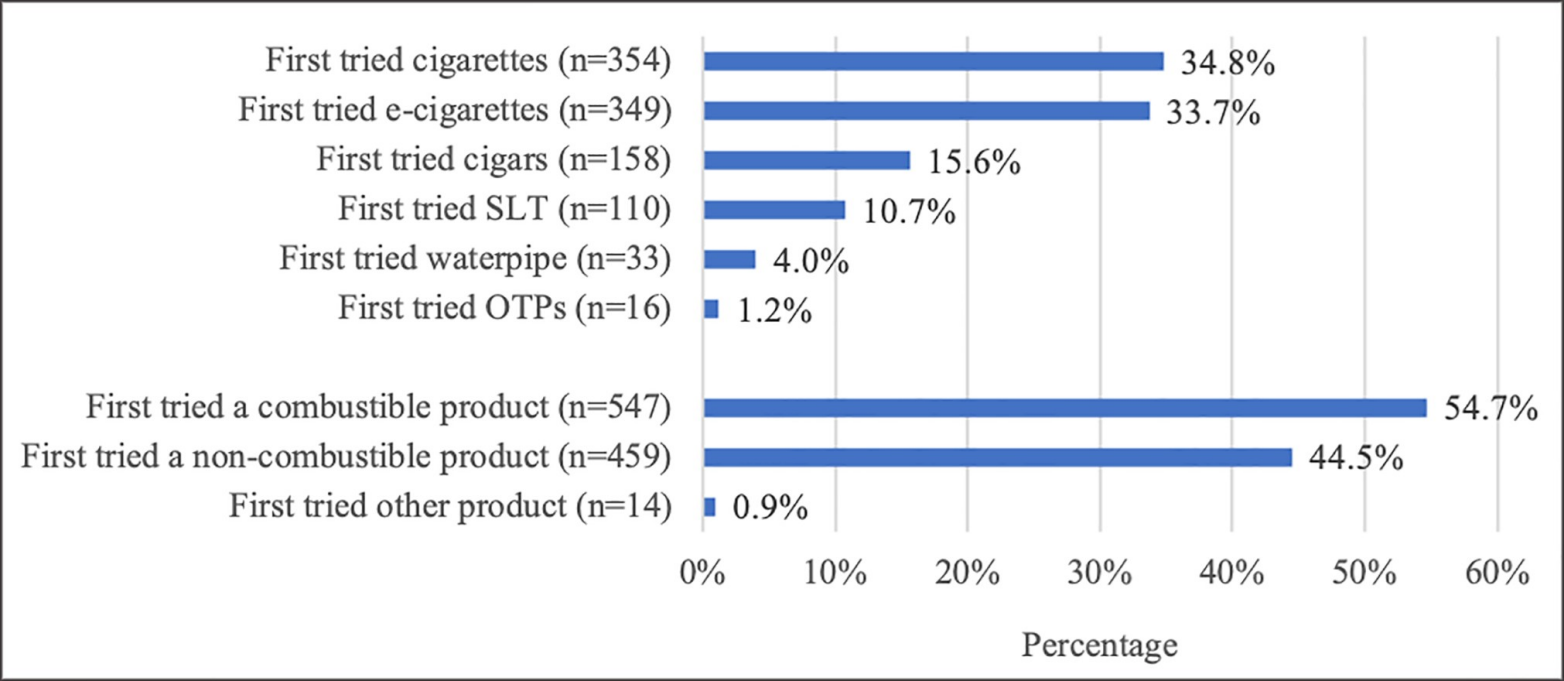
Prevalence of first product tried among ever tobacco product users, n = 1020, North Carolina Youth Tobacco Survey, 2017 Abbreviations: Smokeless tobacco (SLT); Other tobacco products (OTPs), which refers to pipe tobacco or some other tobacco product not listed.

Almost as many adolescents reported e-cigarettes (33.7%) were their first tobacco product tried as adolescents who reported cigarettes (34.8%) were their first tobacco product tried (Fig 1). This was followed by cigars (15.6%), SLT (10.7%), waterpipe (4.0%), and OTPs (including pipe tobacco and other products not listed, 1.2%).

### Research Question 1—Bivariate results: Demographics and first product tried

Sex, race, free or reduced-price lunch, exposure to tobacco advertising via the Internet, perceived risk of tobacco use (‘all tobacco products are dangerous’), secondhand smoke exposure, and living with a tobacco product user were all associated with first product tried (p < 0.10), while grade, exposure to tobacco advertising via retail locations, and perceived risk of tobacco use (‘breathing smoke from other people’s cigarettes or other tobacco products is harmful’) were not significantly associated with first product tried. See S2 Table for full results from the bivariate analyses.

### Research Question 1—Multivariable logistic regression results: Demographics and first product tried

#### Males versus females

Males, relative to females, had lower odds of initiating tobacco use via cigarettes (aOR: 0.62, 95% CI: 0.42, 0.92) and e-cigarettes (aOR: 0.54, 95% CI: 0.41, 0.71), but greater odds of initiating tobacco use via SLT (aOR: 10.29, 95% CI: 4.02, 26.31) (Table 2).

**Table 2:** Adjusted logistic regression results—Demographics and first product tried, among ever tobacco users, n = 1020, North Carolina Youth Tobacco Survey, 2017.

	First tried cigarettes vs. all other products	First tried e-cigarettes vs. all other products	First tried cigars vs. all other products	First tried SLT vs. all other products	First tried waterpipe vs. all other products	First tried OTPs (pipe tobacco, other) vs. all other products
	aOR (95% CI)	aOR (95% CI)	aOR (95% CI)	aOR (95% CI)	aOR (95% CI)	aOR (95% CI)
Male (ref = female)	**0.62 (0.42, 0.92)**	**0.54 (0.41, 0.71)**	1.58 (0.96, 2.60)	**10.29 (4.02, 26.31)**	1.28 (0.37, 4.36)	0.42 (0.11, 1.65)
Non-Hispanic Black (ref = non-Hispanic White)	**0.55 (0.33, 0.91)**	**0.52 (0.32, 0.83)**	**6.17 (3.75, 10.14)**	**0.14 (0.04, 0.44)**	2.57 (0.90, 7.39)	1.43 (0.35, 5.93)
Hispanic (ref = non-Hispanic White)	0.89 (0.63, 1.27)	1.18 (0.79, 1.77)	1.04 (0.55, 1.97)	**0.41 (0.18, 0.92)**	**3.46 (1.38, 8.68)**	1.17 (0.24, 5.74)
Non-Hispanic other race (ref = non-Hispanic White)	0.89 (0.44, 1.82)	0.97 (0.39, 2.45)	0.87 (0.38, 2.02)	1.73 (0.55, 5.44)	—	1.99 (0.30, 13.02)
Free or reduced-price lunch (ref = no)	**2.36 (1.39, 4.01)**	**0.39 (0.27, 0.58)**	1.09 (0.62, 1.93)	1.15 (0.75, 1.76)	0.55 (0.22, 1.33)	2.88 (0.69, 11.99)
Exposed to tobacco advertising via the Internet (ref = not exposed)	0.97 (0.68, 1.37)	**1.28 (1.00, 1.65)**	**0.57 (0.36, 0.90)**	0.89 (0.60, 1.30)	2.63 (0.85, 8.11)	0.72 (0.21, 2.52)
Perceived risk: Agreed that “all tobacco products are dangerous” (ref = no)	**1.64 (1.13, 2.38)**	0.82 (0.60, 1.11)	**0.62 (0.39, 0.99)**	0.91 (0.48, 1.72)	1.15 (0.53, 2.48)	0.47 (0.12, 1.93)
Exposed to secondhand smoke exposure (ref = not exposed)	1.01 (0.71, 1.43)	**0.54 (0.40, 0.75)**	1.23 (0.70, 2.18)	**2.71 (1.60, 4.59)**	2.35 (0.87, 6.34)	**0.17 (0.05, 0.62)**
Living with a tobacco product user (ref = no)	**1.94 (1.38, 2.71)**	**0.54 (0.41, 0.71)**	1.22 (0.82, 1.82)	0.88 (0.51, 1.50)	0.73 (0.34, 1.58)	0.48 (0.19, 1.24)

Note: Boldface indicates p<0.05.

#### Race / Ethnicity

Non-Hispanic Black adolescents, relative to non-Hispanic White adolescents had lower odds of initiating tobacco product use via cigarettes (aOR: 0.55, 95% CI: 0.33, 0.91), e-cigarettes (aOR: 0.52, 95% CI: 0.32, 0.83), and SLT (aOR: 0.14, 95% CI: 0.04, 0.44), but greater odds of initiating tobacco product use via cigars (aOR: 6.17, 95% CI: 3.75, 10.14). In addition, Hispanic adolescents, relative to non-Hispanic White adolescents, had lower odds of initiating tobacco use via SLT (aOR: 0.41, 95% CI: 0.18, 0.92), but greater odds of initiating tobacco product use via waterpipe (aOR: 3.46, 95% CI: 1.38, 8.68).

#### Free or reduced-price lunch

Adolescents on free or reduced-price lunch, relative to those not on free or reduced-price lunch, had lower odds of initiating tobacco product use via e-cigarettes (aOR: 0.39, 95% CI: 0.27, 0.58) but greater odds of initiating tobacco product use via cigarettes (aOR: 2.36, 95% CI: 1.39, 4.01).

#### Tobacco advertising via the Internet

Adolescents who were exposed to tobacco advertising via the Internet, relative to those not exposed, had lower odds of initiating tobacco product use via cigars (aOR: 0.57, 95% CI: 0.36, 0.99) but greater odds of initiating tobacco product use via e-cigarettes (aOR: 1.28, 95% CI: 1.00, 1.65).

#### Perceived risk of tobacco products

Adolescents who agreed that all tobacco products are dangerous, relative to those who did not agree, had lower odds of initiating tobacco product use via cigars (aOR: 0.62, 95% CI: 0.39, 0.99) but greater odds of initiating tobacco product use via cigarettes (aOR: 1.64, 95% CI: 1.13, 2.38).

#### Secondhand smoke exposure

Adolescents who were exposed to secondhand smoke, relative to those who were not exposed, had lower odds of initiating tobacco product use via e-cigarettes (aOR: 0.54, 95% CI: 0.40, 0.75) but greater odds of initiating tobacco product use via SLT (aOR: 2.71, 95% CI: 1.60, 4.59).

#### Living with a tobacco product user

Adolescents who lived with a tobacco product user, relative to those who did not, had lower odds of initiating tobacco product use via e-cigarettes (aOR: 0.54, 95% CI: 0.41, 0.71) but greater odds of initiating tobacco product use via cigarettes (aOR: 1.94, 95% CI: 1.38, 2.71).

### Research Question 2—Bivariate results: First product tried and current tobacco product use

To answer our second research question, we descriptively examined what products adolescents were currently using and stratified results by first product tried (Table 3). In the total sample, 64.6% of adolescents who reported ever trying a tobacco product reported past 30 days use of a tobacco product, with prevalence estimates ranging from 36.7% to 80.4% for each product. In addition, between 14.9% and 50.2% of adolescents first trying any tobacco product currently used multiple tobacco products. The prevalence of current multiple tobacco product use was lowest for adolescents who reported first trying waterpipe (14.9%) and highest for adolescents who reported first trying SLT (50.2%).

**Table 3:** Patterns of Current tobacco product use stratified by first product tried, among ever tobacco users, n = 1020, North Carolina Youth Tobacco Survey ^a^^,^^b^^,^^c^, 2017.

	Total	First tried cigarettes	First tried e-cigarettes	First tried cigars	First tried SLT	First tried waterpipe	First tried OTPs (pipe tobacco, other)
	N (%)	N (%)	N (%)	N (%)	N (%)	N (%)	N (%)
No current tobacco product user, n = 380	380 (35.4)	145 (38.7)	145 (39.0)	47 (24.2)	22 (20.8)	16 (63.3)	5 (19.6)
Single current tobacco product user, n = 317							
Currently using cigarettes only	40 (4.0)	29 (9.0)	6 (1.4)	1 (0.3)	2 (1.7)	1 (1.8)	1 (7.2)
Currently using cigars only	62 (7.7)	11 (2.5)	6 (3.4)	41 (35.4)	0 (0)	6 (9.9)	4 (20.5)
Currently using e-cigarettes only	182 (19.0)	33 (15.2)	120 (33.5)	10 (5.2)	12 (10.7)	0 (0)	1 (8.0)
Currently using SLT only	19 (2.5)	4 (1.2)	2 (0.5)	0 (0)	12 (16.5)	1 (3.0)	0 (0)
Currently using waterpipe only	9 (0.8)	3 (0.6)	2 (0.4)	1 (0.7)	0 (0)	2 (7.1)	1 (6.4)
Currently using OTPs only	0 (0)	0 (0)	0 (0)	0 (0)	0 (0)	0 (0)	0 (0)
Multiple current tobacco product user (using 2 products, n = 142)	142 (14.6)	52 (14.2)	44 (15.1)	24 (16.0)	18 (15.5)	3 (7.5)	1 (14.3)
Multiple current tobacco product user (using 3 or more products, n = 186)	186 (15.9)	77 (18.6)	24 (6.7)	34 (18.3)	44 (34.7)	4 (7.3)	3 (24.0)
Total	1020 (100)	354 (100)	349 (100)	158 (100)	110 (100)	33 (100)	16 (100)

^a^ Cells with less than 50 participants should be interpreted with caution.

^b^ Percentages provided are column percentages, i.e., the percentage in the cell included in the first column and first row indicates that 35.4% of adolescents who reported first trying cigarettes currently do not use any tobacco products.

^c^ Categories are mutually exclusive and all percentages add up to 100

### Research Question 2—Multivariable multinomial logistic regression results: First product tried and current tobacco use

Controlling for demographics and relative to initiating tobacco product use via cigarettes, adolescents who initiated tobacco product use via cigars had higher odds of being a single current tobacco product user (aOR: 2.58, 95% CI: 1.57, 4.25) and higher odds of being a multiple current tobacco product user (aOR: 2.33, 95% CI: 1.31, 4.13) (Table 4). In addition, relative to initiating tobacco product use via cigarettes, adolescents who initiated tobacco product use via SLT (aOR: 2.45, 95% CI: 1.18, 5.04) had higher odds of being a multiple current tobacco product user, whereas adolescents who initiated tobacco product use via e-cigarettes (aOR: 0.57, 95% CI: 0.34, 0.93) had lower odds of being a multiple current tobacco product user.

**Table 4:** Adjusted multinomial logistic regression results—First product tried and current tobacco product use, among ever tobacco users, n = 1020, North Carolina Youth Tobacco Survey, 2017.

	Single current tobacco product use vs. no current use	Multiple current tobacco product use vs. no current use
First tried cigars (ref. first tried cigarettes)	**2.58 (1.57, 4.25)**	**2.33 (1.31, 4.13)**
First tried SLT (ref. first tried cigarettes)	1.66 (0.51, 5.42)	**2.45 (1.18, 5.04)**
First tried e-cigarettes (ref. first tried cigarettes)	1.25 (0.80, 1.95)	**0.57 (0.34, 0.93)**
First tried waterpipe (ref. first tried cigarettes)	0.46 (0.11, 1.99)	0.29 (0.08, 1.02)
First tried OTP (ref. first tried cigarettes)	3.56 (0.96, 13.17)	2.99 (0.70, 12.71)

Note: Boldface indicates p<0.05.

Analyses adjust for all demographic correlates.

### Research Question 3—Bivariate results: First product tried and frequency of tobacco product use

To answer our final research question, we descriptively examined adolescents’ first product tried and frequency of tobacco product use among current tobacco product users (Table 5). Among current tobacco product users, 26.6% (n = 187) currently used at least one product frequently and 73.4% (n = 453) did not currently use any products frequently. The prevalence of frequent tobacco use ranged from a low of 13.9% to a high of 52.0%. In other words, 13.9% of participants who initiated tobacco product use via e-cigarettes currently used at least one tobacco product frequently, while 52.0% of participants who initiated tobacco product use via SLT currently used at least one tobacco product frequently. The prevalence of frequent tobacco product use was higher among multiple current tobacco product users than single current tobacco product users; specifically, among single tobacco product users, 12.7% used one product frequently, whereas among multiple tobacco product users, 42.1% used at least one product frequently (p<0.001) (See S3 Table for these results).

**Table 5:** Bivariate results—First product tried and frequency of tobacco product use, among ever tobacco users, n = 1020, North Carolina Youth Tobacco Survey, 2017 ^a^^,^^b^.

	Non-frequent tobacco product use	Frequent tobacco product use	Total	P-value ^c^
	N (%)	N (%)	N (%)	
First tried cigarettes	140 (67.9)	69 (32.1)	209 (100)	p<0.001
First tried e-cigarettes	169 (86.1)	35 (13.9)	204 (100)	
First tried cigars	84 (78.3)	27 (21.7)	111 (100)	
First tried SLT	37 (48.0)	51 (52.0)	88 (100)	
First tried waterpipe	14 (84.3)	3 (15.7)	17 (100)	
First tried OTPs	9 (72.9)	2 (27.1)	11 (100)	

^a^ Cells with less than 50 participants should be interpreted with caution.

^b^ Percentages provided are column percentages, i.e., the percentage in the cell included in the first column and first row indicates that 67.9% of adolescents who reported first trying cigarettes currently do not use any tobacco products frequently.

c P-value is from an omnibus chi-square test.

### Research Question 3—Multivariable logistic regression results: First product tried and frequency of tobacco product use

Controlling for demographics and relative to initiating tobacco product use via cigarettes (Table 6), we found that adolescents who initiated tobacco product use via SLT had higher odds of currently using at least one tobacco product frequently (aOR: 1.90, 95% CI: 1.04, 3.48) while adolescents who initiated tobacco product use via e-cigarettes had lower odds of currently using at least one tobacco product frequently (aOR: 0.40, 95% CI: 0.23, 0.70).

**Table 6:** Adjusted logistic regression results—First product tried and frequency of tobacco product use, among current tobacco users, n = 640, North Carolina Youth Tobacco Survey, 2017.

	Frequent current tobacco product use vs. non-frequent current tobacco product use
First tried cigars (ref. first tried cigarettes)	0.66 (0.29, 1.59)
First tried SLT (ref. first tried cigarettes)	**1.90 (1.04, 3.48)**
First tried e-cigarettes (ref. first tried cigarettes)	**0.40 (0.23, 0.70)**
First tried waterpipe (ref. first tried cigarettes)	0.44 (0.10, 2.02)
First tried OTP (ref. first tried cigarettes)	0.99 (0.18, 5.44)

Note: Boldface indicates p<0.05.

Analyses adjust for all demographic correlates.

## Discussion

In this study, we found that many adolescents reported initiating tobacco use with emerging tobacco products, such as e-cigarettes, alongside other more traditional tobacco products, such as cigarettes. We also found that trying certain products first, including cigars and SLT, was associated with higher odds of current multiple tobacco product use, that trying SLT was associated with higher odds of current frequent tobacco product use, and that trying e-cigarettes first was associated with lower odds of current multiple tobacco product use. Finally, we found several demographic and psychosocial characteristics, including race/ethnicity and adolescent SES to be associated with first product tried. These results have important implications for public health.

That as many adolescents reported initiating tobacco use with e-cigarettes as cigarettes is novel, and suggests that the tobacco product landscape has markedly shifted, particularly among adolescents. Tobacco prevention efforts need to incorporate increased messaging about multiple emerging tobacco products, including e-cigarettes. While the US Food and Drug Administration (FDA) has begun new communication campaigns about e-cigarettes [25], other efforts to disseminate information about e-cigarettes are needed, especially with the rise of new, popular brands, such as JUUL which contain large amounts of nicotine and have increased exponentially among youth [26, 27].

Interestingly, despite the high number of adolescents who reported trying e-cigarettes first, we found that adolescents who initiated tobacco use via e-cigarettes (relative to cigarettes) had lower odds of current multiple tobacco product use and lower odds of frequently using at least one tobacco product. This finding aligns with previous research showing that adolescents who exclusively use e-cigarettes come from low-risk backgrounds (e.g., they are less likely to have used alcohol, marijuana, or other drugs, they have a lower propensity for sensation seeking) [28]. It is therefore, not surprising, that youth who first tried e-cigarettes had lower odds of being a current multiple tobacco product user or of frequently using tobacco products. However, this does not mean that youth who try e-cigarettes are at low risk for smoking onset in prospective analyses. Indeed, other studies have found that youth who try e-cigarettes are at future risk for using other combustible tobacco products, compared to those who have not tried e-cigarettes [14–18]. Moreover, it should be noted that in our study, one out of every five adolescents in our study who first tried e-cigarettes reported using two or more products in the past 30 days, which suggests that e-cigarettes are not without risks of multiple tobacco product use. Our findings suggest that educational messages could be tailored to lower-risk adolescents, in addition to higher-risk groups (such as adolescents from low SES backgrounds) to avert an increase in adolescent smoking. In addition, longitudinal study designs and nationally representative samples are needed to better understand how youth transition from experimenting with tobacco products to using multiple tobacco products over time and differentiate risk factors for why some adolescent e-cigarette users transition to using multiple tobacco products and others do not.

We also found that adolescents who initiated tobacco use via cigars and SLT (relative to cigarettes) had higher odds of current multiple tobacco product use and that adolescents who initiated tobacco use via SLT had higher odds of currently using at least one tobacco product frequently. Both findings are novel. While fewer adolescents initiated tobacco use via cigars or SLT compared to cigarettes or e-cigarettes, more than a quarter of adolescents in this sample (26.3%) tried these products first, which translates into more than 122,000 high school students in NC alone. Moreover, 34.3% and 50.2% of adolescents who had first tried cigars and SLT, respectively, had transitioned to multiple tobacco product use and 52.0% of adolescents who had first tried SLT transitioned to using at least one tobacco product frequently (i.e., using a product for at least 20 days in the past month). In a previous study of adolescents in young adults, first use of a non-combustible tobacco product (i.e., SLT and e-cigarettes) was associated with higher risk of multiple tobacco product use than a combustible tobacco product (i.e., cigarettes, cigars, waterpipe) [8]. However, results were not differentiated by product so it is impossible to determine whether SLT or e-cigarettes were responsible for increased risk for multiple tobacco product use [8]. It is possible that youth who try cigars and SLT first are more likely to experiment with other tobacco products and supplement their tobacco use compared to youth who try cigarettes first. Future research should explore why youth who had initiated tobacco use via cigars and SLT were at greater risk for current multiple tobacco use and why youth who initiated tobacco use via SLT were at greater risk for currently using at least one tobacco product frequently.

Previous research has documented disparities in tobacco use by race and SES, with African American adolescents being more likely to use cigars [29] and lower SES adolescents being more likely to use cigarettes [30]. Our results extend findings by showing that African American adolescents frequently try cigars and that adolescents of lower SES frequently try cigarettes as their first products. Consequently, interventions and policies to reduce lower SES adolescents’ and African American adolescents’ access to and availability of cigarettes and cigars, respectively, are needed to lessen existing tobacco use disparities. For instance, FDA’s current *Fresh Empire* campaign focuses on at-risk multicultural youth ages 12–17 who identify with hip-hop culture, specifically African American, Hispanic, and Asian American/ Pacific Islander youth [31]. While campaign messages currently focus on cigarettes or highlight risks of any tobacco use [31], opportunities exist for broadening message scope to specifically discuss other tobacco products, such as cigars or waterpipe which African American and Hispanic youth had odds of trying first in this study.

We also found a number of psychosocial correlates to be associated with first product tried. For instance, adolescents who were exposed to secondhand smoke and lived with a tobacco product user had lower odds of initiating tobacco use via e-cigarettes. These findings align with previous research suggesting that youth who use e-cigarettes come from lower-risk backgrounds [28]. In addition, adolescents exposed to secondhand smoke had higher odds of initiating tobacco use via SLT; adolescents who lived with a tobacco product user had higher odds of initiating tobacco use via cigarettes; and adolescents who were exposed to tobacco advertising via the Internet had higher odds of initiating tobacco use via e-cigarettes. These findings suggest mechanisms through which tobacco interventions can prevent and reduce tobacco use. For instance, interventions designed to reduce youth SLT use could focus on reducing exposure to other tobacco product users by targeting regulations surrounding tobacco use in public places or interventions designed to reduce youth cigarette use could provide smoking cessation resources for family members. Moreover, interventions designed to reduce *any* tobacco use could target all of these mechanisms (e.g., exposure to tobacco advertising, perceived risk, exposure to secondhand smoke, and living with tobacco product users) using multilevel intervention designs.

### Limitations

We acknowledge several limitations. First, the survey did not assess age at which high school students first used tobacco products to verify which product adolescents tried first or reasons for first trying products. It is possible, for instance, that students first tried a product within the past 30 days or that they first tried a product several years ago. However, research from 2014–2016 suggests that most youth have tried tobacco products between the ages of 12–14 [32]. Given the age range of high school students in our study (typically between 13–18), it is likely that for many students, their first product tried was not in the past 30 days. Future research using additional measures to assess first product tried should be conducted. Second, all data are self-reported, which may have produced biased results. Third, all data are cross-sectional and we could not examine prospectively how first product tried was associated with tobacco product use. Fourth, while the use of free or reduced-price lunch has been used previously as a marker of adolescent SES [22], many schools and districts are moving towards providing all students free lunch for their students regardless of income. Fifth, all results are from high school students in North Carolina and findings may not generalize to other states or populations. Finally, it is possible that students included in the final sample with complete data differed from those not included in the final sample. Indeed, in an attrition analysis (see S1 Table), we found that a higher proportion of males, non-white adolescents, and non-current tobacco product users were not included in the final sample, either due to missing data or inconsistent data across the different study questions.

## Conclusions

In this study, most adolescents reported initiating tobacco use via cigarettes or e-cigarettes and trying certain products first (e.g., smokeless tobacco) was associated with higher odds of multiple current tobacco product use and higher odds of currently using at least one tobacco product frequently. Examining how youth initiate and experiment with tobacco product use suggest that new efforts focused on emerging tobacco products, such as e-cigarettes, are needed, but that they should not replace existing efforts focused on preventing and reducing use of traditional tobacco products, such as cigarettes, cigars, waterpipe, and smokeless tobacco.

## Supporting information

S1 Table Attrition analysis. (DOCX)

S2 Table Bivariate results—Demographic and psychosocial correlates and first product tried. (DOCX)

S3 Table Bivariate results—Frequency of tobacco product use and current tobacco product use. (DOCX)

S1 Data Data (CSV File). (CSV)
