# Supplementary material for: Adolescents’ first tobacco products: Associations with current multiple tobacco product use
Source: PLoS One. 2019 May 23;14(5):e0217244. doi: 10.1371/journal.pone.0217244 (PMC6532893; doi:10.1371/journal.pone.0217244)
Supplement: S1 Table — (DOCX) [file pone.0217244.s001.docx]

**S1 Table. Attrition Analysis, n=3133, North Carolina Youth Tobacco Survey, 2017**

|  | Not included in final sample, n=2113 (67.4%) | Included in final sample, n=1020 (32.6%) | P-value |
| --- | --- | --- | --- |
| Sex |  |  |  |
| Female | 1027 (69.7%) | 492 (30.3%) | p=0.05 |
| Male | 1080 (70.2%) | 528 (29.8%) |  |
| Grade |  |  |  |
| 9^th^ | 713 (77.9) | 253 (22.1) | p=0.0.005 |
| 10^th^ | 502 (69,8) | 261 (30.2) |  |
| 11^th^ | 430 (67.1) | 211 (32.9) |  |
| 12^th^ | 442 (61.6) | 295 (38.4) |  |
| Race |  |  |  |
| Non-Hispanic White | 1003 (62.7) | 612 (37.3) | p<0.001 |
| Non-Hispanic Black | 534 (79.4) | 190 (20.6) |  |
| Hispanic | 431 (77.3) | 163 (22.7) |  |
| Non-Hispanic other race | 133 (71.9) | 55 (28.1) |  |
| Free or reduced-price lunch |  |  |  |
| Yes | 1012 (69.1) | 496 (30.9) | p=0.59 |
| No | 1080 (70.4) | 524 (29.6) |  |
| Exposure to tobacco advertising via the Internet |  |  |  |
| No | 1124 (71.2) | 514 (28.8) | p=0.05 |
| Yes | 922 (67.2) | 506 (32.8) |  |
| Exposure to tobacco advertising via retail locations |  |  |  |
| No | 561 (81.1) | 177 (18.9) | p<0.001 |
| Yes | 1515 (65.5) | 843 (34.5) |  |
| Perceived risk: Agreed that “all tobacco products are dangerous” |  |  |  |
| No | 849 (59.8) | 664 (40.2) | p<0.001 |
| Yes | 1187 (78.9) | 356 (21.1) |  |
| Perceived risk: Agreed that “breathing smoke from other people’s cigarettes or other tobacco products is harmful” |  |  |  |
| No | 886 (64.0) | 541 (36.0) | p<0.001 |
| Yes | 1190 (74.9) | 479 (25.1) |  |
| Exposed to secondhand smoke exposure |  |  |  |
| No | 1347 (74.5) | 525 (25.5) | p<0.001 |
| Yes | 725 (62.0) | 495 (38.0) |  |
| Living with a tobacco product user |  |  |  |
| No | 1292 (78.5) | 422 (21.5) | p<0.001 |
| Yes | 760 (57.8) | 598 (42.2) |  |
| Current multiple tobacco product use |  |  |  |
| No current use | 1755 (84.8) | 380 (15.2) | p<0.001 |
| Single current use | 92 (19.2) | 312 (80.8) |  |
| Multiple current use | 54 (14.1) | 328 (85.9) |  |
| Frequency of any tobacco product use among current users of any tobacco product |  |  |  |
| Non-frequent | 106 (17.0) | 453 (83.0) | p=0.90 |
| Frequent | 40 (16.5) | 187 (83.5) |  |
| ^b^ Percentages provided are row percentages, i.e., the percentage in the cell included in the first column and first row indicates that among females, 69,7% were not included in the final sample, either due to missing data or because they did not meet the inclusion criteria for the study. | | | |
