# Supplementary material for: Adolescents’ first tobacco products: Associations with current multiple tobacco product use
Source: PLoS One. 2019 May 23;14(5):e0217244. doi: 10.1371/journal.pone.0217244 (PMC6532893; doi:10.1371/journal.pone.0217244)
Supplement: S2 Table — (DOCX) [file pone.0217244.s002.docx]

**S2 Table. Bivariate Results—Demographic and Psychosocial Correlates and First Product Tried, among Ever Tobacco Users, n=1020, North Carolina Youth Tobacco Survey, 2017 ^a,b^**

|  | First tried cigarettes | First tried e-cigarettes | First tried cigars | First tried SLT | First tried waterpipe | First tried OTPs (pipe tobacco, other) | Total | P-value ^c^ |  |
| --- | --- | --- | --- | --- | --- | --- | --- | --- | --- |
|  | N (%) | N (%) | N (%) | N (%) | N (%) | N (%) | N (%) |  |  |
| Sex |  |  |  |  |  |  |  |  |  |
| Female | 189 (39.7) | 194 (39.1) | 66 (12.7) | 11 (2.8) | 21 (4.0) | 11 (1.5) | 492 (100) | p<0.001 |  |
| Male | 165 (30.0) | 155 (28.5) | 92 (18.4) | 99 (18.4) | 12 (3.9) | 5 (0.8) | 528 (100) |  |  |
| Grade |  |  |  |  |  |  |  |  |  |
| 9^th^ | 77 (30.1) | 102 (40.8) | 38 (14.3) | 27 (12.2) | 4 (1.4) | 5 (1.1) | 253 (100) | p=0.24 |  |
| 10^th^ | 99 (33.9) | 101 (38.8) | 29 (11.4) | 19 (8.4) | 8 (6.4) | 5 (1.0)) | 261 (100) |  |  |
| 11^th^ | 77 (36.2) | 65 (32.9) | 35 (15.6) | 25 (10.8) | 7 (3.3) | 2 (1.2) | 211 (100) |  |  |
| 12^th^ | 101 (37.8) | 81 (24.4) | 56 (20.5) | 39 (11.8) | 14 (4.2) | 4 (1.4) | 295 (100) |  |  |
| Race |  |  |  |  |  |  |  |  |  |
| Non-Hispanic White | 214 (35.2) | 232 (37.5) | 60 (10.3) | 90 (13.1) | 12 (3.1) | 4 (0.8) | 612 (100) | p=0.08 |  |
| Non-Hispanic Black | 59 (29.5) | 37 (20.6) | 71 (40.4) | 5 (2.2) | 10 (5.4) | 8 (2.0) | 190 (100) |  |  |
| Hispanic | 58 (35.6) | 64 (36.4) | 18 (10.4) | 9 (7.0) | 11 (9.1) | 3 (1.4) | 163 (100) |  |  |
| Non-Hispanic other race | 23 (44.1) | 16 (28.3) | 9 (8.4) | 6 (16.8) | 0 (0) | 1 (2.4) | 55 (100) |  |  |
| Free or reduced-price lunch |  |  |  |  |  |  |  |  |  |
| Yes | 207 (44.6) | 124 (22.1) | 95 (18.9) | 41 (9.5) | 16 (3.1) | 13 (1.8) | 496 (100) | p<0.001 |  |
| No | 147 (26.5) | 225 (43.6) | 63 (12.8) | 69 (11.8) | 17 (4.7) | 3 (0.6) | 524 (100) |  |  |
| Exposed to tobacco advertising via the Internet |  |  |  |  |  |  |  |  |  |
| No | 175 (33.4) | 167 (31.7) | 91 (19.5) | 61 (11.8) | 13 (2.3) | 7 (1.4) | 514 (100) | p=0.009 |  |
| Yes | 179 (36.3) | 182 (35.8) | 67 (11.6) | 49 (9.7) | 20 (5.7) | 9 (5.7) | 506 (100) |  |  |
| Exposed to tobacco advertising via retail locations |  |  |  |  |  |  |  |  |  |
| No | 70 (39.2) | 54 (30.6) | 30 (16.4) | 11 (6.4) | 8 (5.5) | 4 (1.9) | 177 (100) | p=0.38 |  |
| Yes | 284 (34.0) | 295 (34.3) | 128 (15.4) | 99 (11.6) | 25 (3.7) | 12 (1.0) | 843 (100) |  |  |
| Perceived risk: Agreed that “all tobacco products are dangerous” |  |  |  |  |  |  |  |  |  |
| No | 214 (31.0) | 230 (34.9) | 110 (17.4) | 79 (11.4) | 18 (3.9) | 13 (1.4) | 664 (100) | p=0.08 |  |
| Yes | 140 (42.0) | 119 (31.4) | 48 (12.2) | 31 (9.6) | 15 (4.1) | 3 (0.7) | 356 (100) |  |  |
| Perceived risk: Agreed that “breathing smoke from other people’s cigarettes or other tobacco products is harmful” |  |  |  |  |  |  |  |  |  |
| No | 190 (35.1) | 178 (32.5) | 90 (18.1) | 63 (9.5) | 14 (3.8) | 6 (1.1) | 541 (100) | p=0.33 |  |
| Yes | 164 (34.5) | 171 (35.4) | 68 (12.3) | 47 (12.4) | 19 (4.2) | 10 (1.2) | 479 (100) |  |  |
| Secondhand smoke exposure |  |  |  |  |  |  |  |  |  |
| No | 183 (33.1) | 190 (38.5) | 79 (16.0) | 48 (8.1) | 13 (2.4) | 12 (2.0) | 525 (100) | p=0.001 |  |
| Yes | 171 (36.6) | 159 (28.8) | 79 (15.2) | 62 (13.5) | 20 (5.5) | 4 (0.3) | 495 (100) |  |  |
| Living with a tobacco product user |  |  |  |  |  |  |  |  |  |
| No | 108 (25.2) | 179 (44.5) | 68 (14.3) | 40 (9.5) | 19 (4.6) | 8 (1.8) | 422 (100) | p<0.001 |  |
| Yes | 246 (41.2) | 170 (26.6) | 90 (16.4) | 70 (11.6) | 14 (3.5) | 8 (0.8) | 598 (100) |  |  |
| ^a^ Cells with less than 50 participants should be interpreted with caution.  ^b^ Percentages provided are row percentages, i.e., the percentage included in the cell in the first column and first row indicates that 39.7% of females reported trying cigarettes as their first tobacco product.  ^c^ p-value refers to bivariate results and was calculated using an omnibus chi-square test. Results refer to whether first product tried differs by demographic and tobacco-use variables. | | | | | | | | | |
