# Supplementary material for: Adolescents’ first tobacco products: Associations with current multiple tobacco product use
Source: PLoS One. 2019 May 23;14(5):e0217244. doi: 10.1371/journal.pone.0217244 (PMC6532893; doi:10.1371/journal.pone.0217244)
Supplement: S3 Table — (DOCX) [file pone.0217244.s003.docx]

**S3 Table. Bivariate Results—Frequency of Tobacco Product Use and Current Tobacco Product Use, among Ever Tobacco Users, n=1020, North Carolina Youth Tobacco Survey, 2017 ^a,b^**

|  | Frequency of tobacco product use | | |  |  |
| --- | --- | --- | --- | --- | --- |
| Current tobacco product use | 0 products | 1 product | 2 or more products | Total | P-value ^c^ |
| No use | 0 (0) | 0 (0) | 0 (0) | 0 (100) | p<0.001 |
| Single product use | 276 (87.3) | 36 (12.7) | 0 (0) | 312 (100) |  |
| Multiple product use | 177 (57.9) | 103 (30.4) | 48 (11.7) | 328 (100) |  |
| ^a^ Cells with less than 50 participants should be interpreted with caution.  ^b^ Percentages provided are row percentages, i.e., the percentage included in the cell in the first column and first row indicates that 0% of non-current tobacco product users reported trying 0 products frequently in the past 30 days.  ^c^ p-value was calculated using an omnibus chi-square test. Results refer to whether frequency of tobacco product use differs by current tobacco product use. | | | | | |
